# Psychometric Properties of the Experience of Cognitive Intrusion of Pain (ECIP) Scale in Pediatric Chronic Pain

**DOI:** 10.3390/children12081069

**Published:** 2025-08-14

**Authors:** Cherish Heard, Keri R. Hainsworth, Kristen E. Jastrowski Mano

**Affiliations:** 1 Department of Psychology, College of Arts & Sciences, University of Cincinnati, Cincinnati, OH 45221, USA; heardch@mail.uc.edu; 2 Children’s Wisconsin, Milwaukee, WI 53226, USA; khainsworth@childrenswi.org

**Keywords:** chronic pain, pediatric, validation, cognitive intrusion

## Abstract

**Background/Objectives**: Chronic pain symptoms can disrupt cognitive processes. Such interruptions may negatively impact one’s overall functioning, causing frustration and distress when engaging in important tasks. This experience has been referred to as *cognitive intrusion of pain*. To date, only one adult self-report measure of cognitive intrusion of pain exists: the Experience of Cognitive Intrusion of Pain (ECIP). The purpose of the current study was to examine the psychometric properties of the ECIP in a sample of pediatric patients with chronic pain. **Methods**: The internal consistency reliability, factor structure, and validity of the ECIP were evaluated in a sample (N = 182) of youth ages 11 to 18 who presented to a multidisciplinary chronic pain clinic at a large Midwestern children’s hospital in the United States. **Results**: Results suggest excellent reliability (α = 0.94). Confirmatory factor analysis results supported a one-factor model, with excellent model fit. The ECIP demonstrated evidence of convergent validity, with moderate and positive correlations with measures of pain-related limitations in functioning, pain symptoms, anxiety, and depression. Regarding discriminant validity evidence, the ECIP was minimally and inversely related to measures of readiness to transition to self-managed care and global health. **Conclusions**: Overall, the ECIP demonstrated strong initial reliability and validity evidence for use in pediatric chronic pain. Further research is recommended in more diverse samples and to evaluate the clinical utility of the ECIP.

## 1. Introduction

Pediatric chronic pain, defined as recurrent or persistent pain lasting a minimum of 3 months [1], is estimated to be experienced by 1 in 5 children and adolescents [2]. Chronic pain conditions include headache, abdominal pain, musculoskeletal pain, and neuropathic pain, with headache being the most commonly reported pediatric pain complaint in the United States [2]. The impact of pediatric chronic pain on functional impairment across physical, social, emotional, and academic domains has been demonstrated [3,4,5,6]. The unique challenges faced by pediatric pain patients often negatively impact their quality of life and are associated with higher levels of depression and anxiety [3,7].

Growing evidence suggests that pediatric chronic pain patients experience additional challenges engaging in activities that are cognitively demanding or require attentional control (e.g., paying attention and participating in school, engaging in self-management tasks) [4,8,9,10]. These everyday activities often require a combination of cognitive skills such as sustained attention and working memory. However, those who experience chronic pain may encounter disruptions in cognitive performance due to difficulty maintaining focus on the task at hand, as their focus may be redirected to the pain symptoms they experience [4,11]. This is consistent with theoretical and experimental work suggesting that when individuals experience pain, the degree to which they can inhibit attention directed toward pain, re-orient attentional resources toward something else, and subsequently maintain the [non-pain] orientation of attention depends heavily on one’s available cognitive resources, such as attentional control and task switching abilities [12,13].

This “balancing act” between maintaining engagement with a particular activity while simultaneously managing pain-related disruption has been referred to as cognitive intrusion of pain (CIP). Specifically, CIP is the extent to which pain interferes with an individual’s thoughts and cognitive processes [11,14]. CIP can greatly impact mood, awareness, and overall functioning [14,15,16]. Of note, CIP can impact aspects of important day-to-day functions. For example, in adults with chronic low back pain, research shows that CIP mediates the relationship between pain catastrophizing and driving outcomes, such as distractibility while driving and frequency of driving violations [15]. Although the disruptive effect of pain on cognitive task performance (e.g., working memory, attentional control) has been demonstrated in studies with both adult and pediatric samples [10,12,17,18], to date, the subjective experience of pain intruding on one’s attention and thoughts has not been assessed in youth with chronic pain. This is a critical gap in our understanding of pain-related cognitive impairments in pediatric populations.

### The Experience of Cognitive Intrusion of Pain

To date, only one measure of CIP has been developed. The *Experience of Cognitive Intrusion of Pain* (ECIP) is an adult self-report questionnaire designed to measure the experience and effects of pain-related disruption of attention [11]. Validation of the measure in adults has suggested good internal consistency reliability and construct validity, as ECIP scores were correlated in expected ways with measures of pain vigilance, pain anxiety, depression, anxiety, stress, and overall health [11,14]. In clinical studies, the ECIP has been shown to predict preoperative surgical fear among adults [19] and to negatively impact pain interference and activities of daily living among adults with musculoskeletal pain [20].

To date, despite evidence of reliability and validity in adults with and without chronic pain, the ECIP has not been validated for use with pediatric chronic pain samples. It is critical to evaluate the psychometric properties of validated adult measures with younger populations to determine their appropriateness for use. It cannot be assumed that the psychometric properties of any measure are equivalent across groups that vary in important domains, such as age or developmental stage [21]. Furthermore, though inquiring about pain-related cognitive dysfunction in adults with chronic pain may be more common given the expected cognitive decline associated with aging, research in pediatric chronic pain has demonstrated that cognitive deficits are present in many younger pain patients. For example, pediatric chronic pain samples show pronounced executive functioning deficits in the areas of working memory, cognitive flexibility, and inhibition compared to healthy peers [4,22,23]. Such executive functioning challenges are linked to difficulties across academic, social, emotional, and physical domains [4].

The purpose of the current study was to examine the psychometric properties of the ECIP in a sample of pediatric patients with chronic pain. Our primary aim was to better understand the presence and impact of pain-related cognitive intrusion through the evaluation of internal consistency reliability, factor structure, and aspects of convergent and discriminant validity of the ECIP in pediatric pain patients.

## 2. Materials and Methods

### 2.1. Participants

The study sample consisted of youth ages 11 to 18 who presented to a multidisciplinary chronic pain clinic at a large Midwestern children’s hospital in the United States between September 2020 and March 2021. To be included in the study, youth needed to be able to read written English.

### 2.2. Procedure

Data were collected as part of a clinical care registry at the Children’s Wisconsin (CW) Pain and Headache Center using the Pediatric adaptation of the Collaborative Health Outcomes Information Registry platform (Peds-CHOIR) [24]. The registry is approved for clinical care by the Medical College of Wisconsin (MCW) Institutional Review Board (IRB) (IRB #25773). CHOIR is a HIPAA-compliant, open-source standard data registry platform. A separate IRB approval was obtained to use data from both Peds-CHOIR and EPIC medical records for this retrospective study (IRB # 50013). Prior to completion of any questionnaires in the Peds-CHOIR platform, all patients ≥13 years (and parents of children ≤12 years) opt in or out of the use of their data for research purposes. Only data from patients who agreed to the use of their clinical data for research were included in this study.

Patients completed questionnaire measures of pain characteristics, demographics, emotional functioning (anxiety and mood), and pain-specific activity limitations as part of the standard intake procedure for the Pain and Headache clinic. Families were asked to complete the questionnaires individually at home up to two weeks before the initial intake appointment. Patients and families without completed data at the time of the appointment were given tablets to complete the questionnaires upon arrival to the clinic. Patient pain diagnoses were documented by an anesthesiologist or Advanced Practice Nurse. Primary pain condition was categorized according to pain location/diagnosis [head (e.g., headaches, migraines), chest/neck/back, extremities (e.g., feet, hands), abdomen (e.g., functional abdominal, gastrointestinal), musculoskeletal, and other]. Participants whose pain diagnoses were not listed were removed from any analyses pertaining to pain condition.

### 2.3. Measures

#### 2.3.1. The Experience of Cognitive Intrusion of Pain (ECIP)

The ECIP is a 10-item self-report measure of the impact of chronic pain on cognitive functioning (e.g., concentrating). The ECIP [11] consists of three subscales (Interruption, Control, and Rumination); however, results of an exploratory factor analysis suggested that the experience of cognitive intrusion is a unitary construct, with all items loading on to a single factor [14]. Total scores range from 0 to 60 with higher scores reflecting greater difficulty with pain-related cognitive intrusion. Among adult chronic pain and healthy control groups, the ECIP has shown high internal consistency reliability (Cronbach’s alpha = 0.96 [11]).

To determine whether wording adaptations would be necessary prior to administering the ECIP to adolescents, four pediatric pain psychologists with research and clinical expertise in chronic pain in children and adolescents reviewed ECIP items. A few wording changes were made to make the items more developmentally appropriate and to avoid terms/expressions that would likely be unfamiliar to younger respondents. For example, the ECIP item “Pain dominates my thinking” was changed to “Pain controls my thinking” and “Pain intrudes on my thoughts” was changed to “Pain disrupts my thoughts.” Further, though a 0 to 6 Likert scale was maintained, we simplified the response options. Specifically, the original ECIP used a 0—*Not at all applicable* to 6—*Highly applicable* scale, which we modified to a 0—*Does not describe me* to 6—*Describes me*. After these minor wording revisions were made, ECIP items were then reviewed for clarity and understanding by two adolescent volunteers (see Appendix A for the modified ECIP).

#### 2.3.2. The Child Activity Limitations Interview (CALI-9)

The CALI-9 is a 9-item, child-self-report measure of the degree to which a child or adolescent experiences pain-related functional limitations [25]. The CALI-9 yields a total score and 2 factors: Active and Routine activity limitations. Among pediatric chronic pain samples, CALI-9 items were significantly positively associated with the previous longer validated version of the measure, the CALI-21 (*r* = 0.95, *p* < 0.001). In the current sample, a reliability analysis revealed good internal consistency (Cronbach’s alpha = 0.81).

#### 2.3.3. The Patient-Reported Outcomes Measurement Information System Scale Anxiety (PROMIS—Pediatric Anxiety)

The PROMIS—Pediatric Anxiety measure was administered using computerized adaptive testing (CAT). It is a child self-report measure assessing fear, anxiety, hyperarousal, and somatic symptoms [26]. Previous research results revealed good convergent validity when compared to the long form (*r* = 0.96) in adult samples. This measure displayed good convergent validity (*r* = 0.72) when compared with other similar constructs, and a confirmatory factor analysis revealed that the measure demonstrated support for the model [27]. Because the measure was administered using CAT and only PROMIS *t*-scores were used in the current study, we could not calculate an item-level internal consistency coefficient. However, results from a systematic review in US pediatric samples have shown high internal consistency (Cronbach’s alpha of 0.93 [28]).

#### 2.3.4. The Patient-Reported Outcomes Measurement Information System Scale Depression (PROMIS—Pediatric Depression)

The PROMIS—Pediatric Depression measure was also administered using CAT. It is an 8-item, child-self-report measure assessing the severity of pediatric depression within the last 7 days [26]. Previous research results revealed good convergent validity when compared to the long form (*r* = 0.96). Results also revealed high internal consistency (Cronbach’s alpha = 0.97), and a confirmatory factor analysis supported a 1-factor model [29] in adult samples. Because the measure was administered using CAT and only PROMIS *t*-scores were used in the current study, we could not calculate an item-level internal consistency coefficient. However, in US pediatric populations, high internal consistency (Cronbach’s alpha = 0.95 [28]) has been demonstrated.

#### 2.3.5. Pain Frequency–Severity–Duration (PFSD) Scale

The PFSD is a self-reported pain questionnaire that assesses multiple aspects of the patient’s pain experience [30]. The PFSD asks respondents to indicate the number of days with pain in the past two weeks (pain frequency), and usual and worst pain intensity over the past week, with both pain intensity responses based on an 11-point Likert scale ranging from 0 to 10 (“No pain” to “Worst pain you can imagine”).

#### 2.3.6. The Patient-Reported Outcomes Measurement Information System Pediatric Global Health Scale (PROMIS—Global Health-7)

The PROMIS—Global Health-7 was administered using CAT. It is a youth self-report measure of health-related quality of life (physical and social health [31]). The PROMIS—Global Health-7 has evidenced acceptable structural and convergent validity (*r* = 0.69) when correlated with other quality of life measures (e.g., PedsQL). Because the measure was administered using CAT and only PROMIS *t*-scores were used in the current study, we could not calculate an item-level internal consistency coefficient, but high internal consistency (Cronbach’s alpha = 0.88 [32]) has been demonstrated in US pediatric populations.

#### 2.3.7. Self-Management and Transition to Adulthood with Rx = Treatment (STARX)

The STARX is a child self-report measure assessing adolescent and young adult readiness to transition to self-management of a chronic condition [33]. This measure consists of 6 factors (Provider Communication, Medication Management, Engagement During Appointments, Disease Knowledge, Adult Health Responsibilities, and Resource Utilization). Previous research revealed good internal consistency (Cronbach’s alpha = 0.80 [34]). In the current sample, Cronbach’s alpha was acceptable (0.65).

### 2.4. Analytic Approach

All data were analyzed using SPSS (Version 30) and MPlus (Version 7.3). Preliminary analyses consisted of examining descriptive statistics of sample characteristics (e.g., participant sex, age, and primary pain condition) as well as ECIP total scores (skewness, kurtosis, and homogeneity) and individual items. Cronbach’s alpha was calculated to estimate the internal consistency reliability of the ECIP. Based on the ECIP validation work in adults [11,14], we hypothesized a one-factor model of cognitive intrusion, which was tested using confirmatory factor analysis (CFA). Correlational analyses were performed to assess the construct validity of the ECIP. Because CIP has been shown to impact overall functioning, mood, and awareness [11,14,15,16], we hypothesized that ECIP scores would demonstrate statistically significant positive correlations with scores from measures of pain-related functional limitations [CALI-9], depression [PROMIS—Depression], anxiety [PROMIS—Anxiety], and pain symptoms [PFSD]; as well as an inverse relationship with global health (i.e., those reporting higher ECIP scores would score lower on the PROMIS—Global Health-7). We also predicted that the ECIP would demonstrate evidence of discriminant validity, with negative, low-magnitude correlations (as indicated by *r* < 0.30) between ECIP scores and a measure of pediatric readiness to transition to self-management of chronic pain symptoms [STARX]. Of the available measures in the clinic registry, the STARX was deemed a reasonable choice for an index of discriminant validity, given that readiness to transition to adult care is theoretically unrelated to CIP. Lastly, *t*-tests and ANOVAs were conducted to explore possible group differences in pain intrusion based on sex and primary pain condition.

## 3. Results

### 3.1. Sample Characteristics

The final sample included 182 participants ages 11 to 18 (M = 14.25, SD = 2.15). Six potential participants were removed from the dataset because they did not complete any self-report questionnaires, including the ECIP. Most participants were female (77.5%) and self-identified as White (81.9%). Table 1 provides detailed sample characteristics pertaining to age, sex, race, ethnicity, and primary pain location.

### 3.2. Preliminary Analyses

Total ECIP score means were calculated by participant sex, age, and primary pain location. Patient age was not significantly correlated with ECIP scores (*r* = −0.04, *p* = 0.59). An independent samples *t*-test indicated there was no significant difference in total ECIP scores between males (M = 30.53, SD = 13.12) and females (M = 30.65, SD = 15.05; t(180) = 0.05, *p* = 0.96). A one-way analysis of variance (ANOVA) revealed no significant differences in overall ECIP scores based on primary pain condition [F(5,174) = 1.04, *p* = 0.398].

Higher ECIP scores indicate higher cognitive intrusion of pain (with 60 as the highest possible total score). A histogram and P-P plot revealed that the data was normally distributed across ECIP items. A test of skewness and kurtosis revealed that scores were within range of a normal distribution. Skewness and kurtosis values were assessed based on the assumption that when 50 < N < 300 (indicating a medium sample size), any value over 3.29 would constitute rejecting the null hypothesis of normality, indicating a non-normal distribution [35]. The Levene’s test revealed that homogeneity was not violated (*p* = 0.053). Table 2 displays the mean, standard deviation, and range of ECIP scores based on participant age, sex, race, ethnicity, and primary pain location.

### 3.3. ECIP Item Analysis

All individual ECIP items contained the full range of scores (0–6) with 0 being “does not describe me” and 6 being “describes me”. ANOVAs revealed no significant mean differences across age for individual ECIP items. Levene’s test revealed no violations of homogeneity across age groups for individual item scores.

### 3.4. Internal Consistency Reliability

A reliability analysis revealed excellent internal consistency of ECIP scores, with a Cronbach’s α of 0.94 for the full sample. When examining reliability within each age group, Cronbach’s α ranged from 0.97 (11-year-olds) to 0.76 (18-year-olds).

### 3.5. Confirmatory Factor Analysis

**Figure 1:**
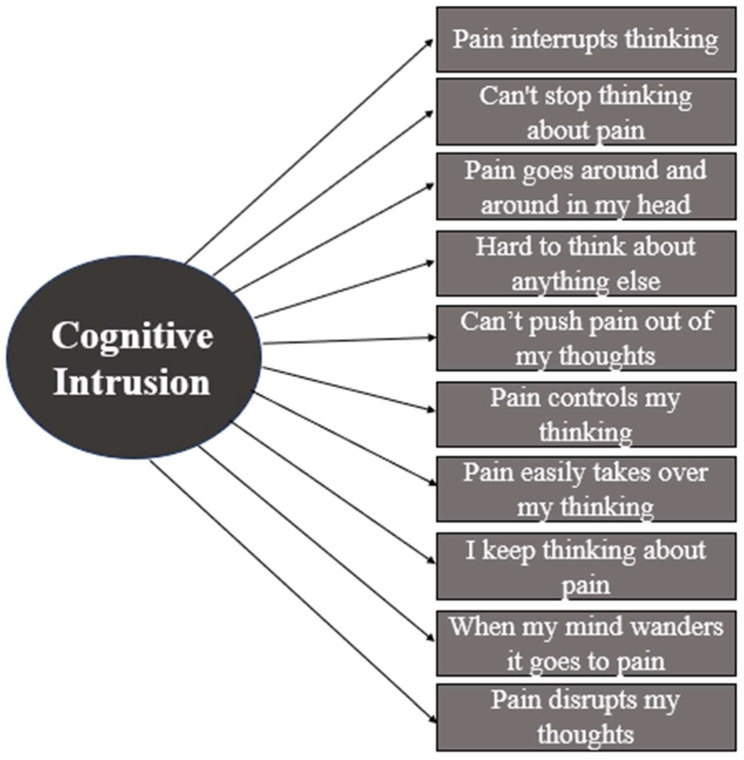
One-factor model of cognitive intrusion as measured by the Experience of Cognitive Intrusion (ECIP) scale [11,14].

A CFA was conducted to test the one-factor model of cognitive intrusion of pain. The measurement scale was defined by means fixed at zero and variances at one for standardization. The CFA supported a one-factor model for the ECIP. All 10 ECIP items (indicators) loaded onto a single latent construct (See Figure 1). The ECIP displayed excellent model fit, *χ*^2^ = 30.24, *df* = 23, *p* = 0.14; CFI = 0.99 (excellent fit), TLI = 0.99 (excellent fit), RMSEA = 0.042 (good fit; 90% CI 0.00–0.078), and SRMR = 0.021 (good fit). Across the collective fit statistics, the model demonstrated good to excellent fit. To determine whether any indicator (item) error terms were highly correlated, we generated a modification index (MI). A MI measures how much a model’s goodness-of-fit (in chi-square units) improves if any two specific error terms are allowed to correlate. The suggested modifications were incorporated to allow for shared measurement error of items (e.g., the error term for item 2 [I can’t stop thinking about the pain] correlated with the error terms for items 3, 4, 6, and 7). This is common practice in CFA if consistent with theory and expected based on the nature of the construct and item characteristics (e.g., questionnaire items with similar wording). Only those MIs that were consistent with theory and expected based on the nature of the ECIP items were considered.

### 3.6. Convergent and Discriminant Validity

ECIP scores and PFSD composite scores were significantly positively correlated, indicating that as multiple aspects of the pain experience worsen, so does cognitive intrusion of pain (*r* = 0.23, *p* = 0.002). As hypothesized, there was a significant positive correlation between ECIP and CALI-9 raw scores (*r* = 0.36, *p* < 0.001). Significant correlations were also found between ECIP scores and PROMIS—Anxiety T-scores (*r* = 0.30, *p* < 0.001), PROMIS—Depression T-scores (*r* = 0.33, *p* < 0.001), and PROMIS Global Health T-scores (*r* = −0.27, *p* < 0.001). The ECIP also demonstrated evidence of discriminant validity, with a negative correlation between ECIP and STARX scores (*r* = −0.24, *p* = 0.005).

## 4. Discussion

The current study examined the psychometric properties of the ECIP to evaluate evidence of reliability and validity among pediatric chronic pain patients. To our knowledge, the ECIP is the only self-report measure developed to assess cognitive intrusion of pain. While the ECIP has been validated for use with adults experiencing chronic pain, the current study was the first to examine the subjective experience of pain intruding on one’s attention and thoughts among younger chronic pain patients (ages 11–18). It is important that the psychometric properties of the ECIP are adequately assessed prior to determining possible clinical utility with pediatric populations. Overall, the findings of this study suggest strong evidence of internal consistency, convergent validity, discriminant validity and factorial validity.

The ECIP demonstrated excellent internal consistency reliability among patients ages 11 to 17, and acceptable reliability for 18-year-olds, suggesting that items are measuring the same construct. Regarding evidence of convergent validity, the ECIP showed expected relationships with theoretically related constructs, such as significant, positive correlations with pain symptoms as well as measures of functional impairment, anxiety, and depression. We also found evidence of discriminant validity via low magnitude, negative associations between ECIP scores and those from measures of transition readiness and global health. Taken together, this pattern of associations suggests that the ECIP is adequately assessing the extent to which pain intrudes upon the cognitive processes of adolescents diagnosed with chronic pain and that the ECIP is capturing something distinct from other measures commonly used with chronic pain patients. The confirmatory factor analysis results supported a one-factor model of cognitive intrusion, such that all ECIP items loaded onto a single latent factor. This is consistent with prior validation work with adult samples [11,14] indicating that cognitive intrusion of pain, as measured using the ECIP, represents a unidimensional construct.

The findings of the current study should be discussed in the context of its limitations. Although this study consisted of a large, representative treatment-seeking sample of pediatric chronic pain patients, it only consisted of patients reporting from one Midwestern hospital in the United States. Possible regional and cultural differences in reporting should be considered. It is also important to acknowledge that nearly two-thirds of participants reported chronic headaches as their primary pain diagnosis. Though headache is the most common pediatric pain complaint in the United States [2], certain clinical phenomena—such as cogniphobia, the fear or avoidance of cognitive exertion due to the belief that it causes headaches [36]—are unique to headache patients. This warrants additional inquiry to examine whether cognitive intrusion is experienced differently as a function of primary pain diagnosis. Further, due to the lack of racial and ethnic representation in the current sample (a common problem in pediatric chronic pain research), possible racial or ethnic differences in reporting could not be meaningfully assessed. Replicating current findings pertaining to the psychometric properties of the ECIP in diverse populations is needed for equitable research and clinical care. While results from the current study may generalize to similar treatment-seeking pediatric chronic pain populations, it is important to reach minoritized populations, including those of lower socioeconomic status, who may have reduced access to medical care due to multisystemic barriers. In past research [37], lower SES was associated with higher pain prevalence. It is possible that similar associations exist between SES and cognitive intrusion of pain.

As far as sex differences, most of the sample was female; thus, potential sex differences in the experience of cognitive intrusion may need to be considered with samples comprising a larger subset of male pediatric pain patients. Future studies should also seek to include more gender-diverse populations. Further, we excluded pediatric patients under the age of 11 given the developmentally advanced nature of the ECIP items and to be consistent with widely used self-report questionnaires for youth assessing aspects of thinking and behavior that usually begin around age 11 or 12 [38]. It is possible that the ECIP, in its current form, may not be psychometrically sound for younger pediatric pain patients, though this should be systematically evaluated in future studies. If there are barriers to reporting cognitive intrusion of pain in younger pediatric populations, a different measure may need to be developed, perhaps relying on parent-proxy reports. Overall, developmental differences should be considered when assessing disrupted cognition in younger samples, in part because chronic pain disrupting key cognitive functions could have an impact on reaching educational and social–emotional developmental milestones.

Future work should also consider whether cognitive intrusion due to pain impacts other key domains of functioning (e.g., interpersonal, physical, and self-care). It is possible that the interruptive effect of pain extends to other areas, such as attending to and processing information during a conversation, and accurately responding. For adolescents, social interactions and social status can be very meaningful and impact overall wellbeing [39]. In general, it may be helpful to understand the additional challenges youth with chronic pain may face when experiencing interruption of attention by pain. Also warranted are examinations of whether ECIP scores relate to cognitive assessments as well as self-report measures of executive functioning that focus on how individuals regulate their attention in everyday situations. In the current study, we did not have information about patient’s baseline neurocognitive processing. Given the relevance of neurodiversity to cognitive functions, subsequent studies would benefit from taking diagnoses of, for example, autism spectrum disorder and attention-deficit hyperactivity disorder into account.

Evaluating the clinical utility of the ECIP is another important area for future research, as consideration of individual differences in cognitive and affective factors contributing to the pain experience is critical to treatment planning and patient care [40]. Research efforts may help to determine the meaning of ECIP scores (e.g., in what ways ECIP scores relate to other clinical variables, such as pain catastrophizing, fear of pain, or treatment readiness) to assist clinicians with case conceptualization. For example, pain-related cognitive intrusion and catastrophizing may overlap in some ways, and both certainly impede optimal engagement in activities of daily living. However, there is evidence for the unique roles played by pain catastrophizing and cognitive intrusion. Among adult patients with musculoskeletal pain, findings suggest that cognitive intrusion and pain catastrophizing distinctly mediate the relationship between pain and interference with activities of daily living [20]. Though we did not assess pain catastrophizing in the current study, its relationship with cognitive intrusion of pain should be further evaluated to establish the clinical utility of the ECIP in pediatric pain. Furthermore, it may also be beneficial to track changes in ECIP scores longitudinally (e.g., at pre- and post-treatment) as one way to assess pain intervention outcomes and improved patient functioning.

While the high internal consistency of the ECIP indicates it is a reliable measure, it may also suggest possible item redundancy. It may be beneficial to explore a shortened version of the ECIP among pediatric samples to account for possible redundancy. Previous studies have shown improved efficiency for measures with fewer items [41]. A shortened measure may maintain high internal consistency and good construct validity, while decreasing patient burden. Also, it may be beneficial to incorporate reverse-scored items in the ECIP to reduce response bias and stimulus generalization.

## 5. Conclusions

The ECIP demonstrated strong reliability and validity evidence in a treatment-seeking sample of pediatric patients diagnosed with a chronic pain condition. Understanding and addressing cognitive intrusion of pain may prove to be an important target for tailored interdisciplinary pain management interventions.

## Figures and Tables

**Table 1:** Sample characteristics (N = 182).

	*M*	*SD*
Age	14.25	2.15
	*n*	(%)
Sex *		
Female	141	77.5
Male	41	22.5
Race		
White/Caucasian	149	81.9
Black/African American	23	12.6
Asian American/Pacific Islander	3	1.6
Multiracial	2	1.1
Preferred Not to Answer	5	2.7
Ethnicity		
Hispanic/Latine/Latino	20	10.9
Not Hispanic/Latine/Latino	160	87.9
Preferred Not to Answer	2	1.1
Primary Pain Location	n	%
Head	117	65
Chest/Neck/Back	10	5.5
Extremities	10	5.5
Abdomen	15	8.3
Musculoskeletal	19	10.5
Other	9	5

* Assigned sex at birth.

**Table 2:** ECIP mean scores by participant age, sex, race, ethnicity, and primary pain location.

	*M*	*SD*	Minimum–Maximum
Full Sample	30.64	14.60	1–60
Age (in years)			
11	33.84	16.67	2–60
12	27.52	14.75	1–53
13	31.57	13.57	2–57
14	31.60	17.36	6–59
15	32.16	14.43	4–60
16	28.07	14.32	1–51
17	30.42	14.28	3–50
18	28.71	7.78	18–38
Sex			
Female	30.65	15.05	1–60
Male	30.53	13.12	1–59
Race			
White/Caucasian	30.25	14.55	1–60
Black/African American	34.91	14.86	7–60
Asian American/Pacific Islander	19.00	13.75	7–34
Multiracial	26.50	13.44	17–36
Preferred Not to Answer	30.80	15.51	9–46
Ethnicity			
Hispanic/Latine/Latino	23.50	15.17	1–56
Not Hispanic/Latine/Latino	31.33	14.26	1–60
Preferred Not to Answer	46.0	18.38	33–59
Primary Pain Location			
Head	30.68	13.62	1–60
Chest/Neck/Back	26.60	16.64	2–53
Extremities	30.50	19.93	1–60
Abdomen	30.33	18.42	4–59
Musculoskeletal	26.95	12.66	4–46
Other	39.33	14.47	7–56

## Data Availability

The data underlying this article cannot be shared publicly due to the privacy of the individuals who participated in the study. The data will be shared on reasonable request by the corresponding author.

## Abbreviations

The following abbreviations are used in this manuscript:

ECIP	Experience of Cognitive Intrusion
CAT	Computerized adaptive testing
PROMIS	Patient-Reported Outcomes Measurement Information System Scale
